# Mutation in collagen II alpha 1 isoforms delineates Stickler and Wagner syndrome phenotypes

**Published:** 2013-04-05

**Authors:** Khanh-Nhat Tran-Viet, Vincent Soler, Valencia Quiette, Caldwell Powell, Tammy Yanovitch, Ravikanth Metlapally, Xiaoyan Luo, Nicholas Katsanis, Erica Nading, Terri L. Young

**Affiliations:** 1 Duke Center for Human Genetics, 905 S LaSalle Street, 27705, Durham, North Carolina; 2 UMRS 563, Centre de Physiopathologie de Toulouse Purpan, Université Paul Sabatier, Toulouse, France; 3 The Department of Ophthalmology, The University of Oklahoma/Dean McGee Eye Institute, Oklahoma City, OK; 4 Departments of Cell Biology and Pediatrics, Center for Human Disease Modeling, Duke University, Durham, NC; 5 The Department of Ophthalmology, Duke University Eye Center, Durham, NC; 6 Vision Science Group, School of Optometry and Departments, University of California, Berkeley, CA

## Abstract

**Purpose:**

Stickler syndrome is an arthro-ophthalmopathy with phenotypic overlap with Wagner syndrome. The common Stickler syndrome type I is inherited as an autosomal dominant trait, with causal mutations in collagen type II alpha 1 (*COL2A1*). Wagner syndrome is associated with mutations in versican (*VCAN*), which encodes for a chondroitin sulfate proteoglycan. A three-generation Caucasian family variably diagnosed with either syndrome was screened for sequence variants in the *COL2A1* and *VCAN* genes.

**Methods:**

Genomic DNA samples derived from saliva were collected from all family members (six affected and four unaffected individuals). Complete sequencing of *COL2A1* and *VCAN* was performed on two affected individuals. Direct sequencing of remaining family members was conducted if the discovered variants followed segregation.

**Results:**

A base-pair substitution (c.258C>A) in exon 2 of *COL2A1* cosegregated with familial disease status. This known mutation occurs in a highly conserved site that causes a premature stop codon (p.C86X). The mutation was not seen in 1,142 ethnically matched control DNA samples.

**Conclusions:**

Premature stop codons in *COL2A1* exon 2 lead to a Stickler syndrome type I ocular-only phenotype with few or no systemic manifestations. Mutation screening of *COL2A1* exon 2 in families with autosomal dominant vitreoretinopathy is important for accurate clinical diagnosis.

## Introduction

Stickler syndrome is a genetically and clinically heterogeneous condition first described in 1965 by Gunnar Stickler as an inherited progressive arthro-ophthalmopathy [1]. Although Stickler syndrome is the most frequently inherited cause of retinal detachment in childhood, this disorder is rare, and the reported prevalence in the United States is 1:10,000 [2-4].

The autosomal dominant (AD) Stickler syndrome subtypes include Stickler syndrome type I (STL1, OMIM 108300), Stickler syndrome type II (STL2, OMIM 604841), and Stickler syndrome type III (STL3, OMIM 184840). STL1 results from altered type II collagen molecules encoded by the collagen type II alpha 1 (*COL2A1*) gene while STL2, caused by mutations in the collagen type XI alpha 1 (*COL11A1*) gene, and STL3, caused by mutations in the collagen type XI alpha 2 (*COL11A2*) gene, affect the type XI collagen molecules [5]. Since 2006, mutations in the collagen type IX alpha 1 (*COL9A1*) and collagen type IX alpha 2 (*COL9A2*) genes, encoding for type IX collagen, have been reported as causal for an autosomal recessive form of Stickler syndrome [6,7].

Type II, IX, and XI collagen molecules are major extracellular matrix components in the hyaline cartilage and the vitreous, and are structurally and functionally associated [8-10]. Affected individuals with mutations in STL1 and STL2 demonstrate a combination of ocular and systemic manifestations. In contrast, STL3 involves only non-ocular manifestations; affected individuals present with systemic malformations such as deafness and cleft palate [7,10-13]. Ocular features associated with AD Stickler syndrome consist of myopia, vitreous degeneration, radial perivascular retinal degeneration, presenile cataract, and high incidence of retinal detachments [14]. The vitreous phenotype depends on the AD Stickler syndrome type, since STL1 vitreous demonstrates a congenital retrolental membrane while patients with STL2 demonstrate vitreous with fibrillar or beaded aspect [15-17]. Non-ocular manifestations of AD Stickler syndrome include arthropathy, hearing impairment, flat midface, midline clefting, and skeletal abnormalities, but these findings can be highly variable, even within a family [5]. Autosomal recessive forms of Stickler syndrome may present with ocular features, such as high myopia and vitreoretinal degeneration but no presenile cataract, and systemic features may include but are not limited to short stature, hearing impairment, facial structural abnormalities but no midline clefting, and causal gene-dependent spondyloepiphyseal dysplasia [7].

The expression of the *COL2A1* gene, associated with STL1, is tissue-dependent, due to an alternative splicing of exon 2, which results in two isoforms of type II collagen [18]. Type IIB is the shorter form with 53 exons (exon 2 spliced), and is mainly expressed in the cartilage while type IIA is the longer form with 54 exons (including exon 2) and is predominantly present in the vitreous [18]. Thus, mutations in *COL2A1* exon 2, such as a premature stop codon, may lead to the ocular variant of the STL1. This ocular condition shares typical STL1 ocular features, such as membranous vitreous and radial perivascular retinal degeneration, but shows none or few systemic manifestations [3,19-21]. To date, several families harboring *COL2A1* exon 2 mutations have been reported [3,19,20,22-25]. In these families, the reported penetrance of vitreoretinal degeneration is more than 90% in affected patients at the age of 20 [19,20].

Wagner syndrome (OMIM 14200) is also associated with the extracellular matrix component gene versican (*VCAN*; alternatively called *CSPG2*), identified in 2005 by Miyamoto et al., which encodes for a large chondroitin sulfate proteoglycan versican [26-30]. We confirmed the involvement of this gene in 2009 by describing an intronic base pair splice site substitution in *VCAN* segregating with the disease in a multigenerational family resulting in a truncated protein affected by the splicing [31]. Wagner syndrome clinical features involve ocular manifestations close to those found in Stickler syndrome. However, patients also complain of nyctalopia, where the vitreous phenotype is different as characterized by an optically empty aspect with avascular strands and veils or fibrillary condensations [32], retinal detachments occur rarely, and retinal features demonstrate retinitis pigmentosa–like “bone spicule” pigmentary atrophy [20] with accompanying electroretinogram abnormalities. Nevertheless, in some cases, distinguishing Wagner syndrome from the ocular-only variant of STL1 may be difficult. Erosive vitreoretinopathy syndrome (OMIM 143200) is another ocular-only disorder, first reported by Brown et al. [33], that can also lead to indeterminate diagnosis based on clinical features only [30]. The clinical features of erosive vitreoretinopathy syndrome include night blindness, visual field defects, and chorioretinal atrophy. The variability in the clinical presentations of vitreoretinal disease phenotypes underscores the importance of careful clinical assessment.

We clinically and genetically report a three-generation Caucasian family from the southeast United States demonstrating AD vitreous degeneration with variable phenotypes among affected members. Over the years, one affected member was initially diagnosed clinically with Stickler syndrome and then Wagner syndrome by her ophthalmologist. We conducted Sanger sequencing of the *COL2A1* (NM_001844) and *VCAN* (NM_004385) genes to delineate the genetic etiology of disease in this family.

## Methods

### Study subjects

Ten individuals (six affecteds, four unaffecteds) from a three-generation Caucasian family was recruited. All consenting family members (four males, six females) were recruited under the approval of the Duke University Institutional Review Board according to the principles of the Declaration of Helenski, under the research protocol entitled “Clinical and Molecular Analysis of Genetic Eye Disorders”, to include molecular genetic testing (protocol number Pro00008040). Individuals underwent ophthalmic examinations that included health histories regarding systemic issues such as cleft palate, midline defects, skeletal or joint abnormalities, and early onset arthritis. The clinical evaluation included assessment tests of Early Treatment Diabetic Retinopathy Study visual acuity (Snellen equivalent) and intraocular pressure, slit-lamp inspection of the anterior segment, and indirect ophthalmoscopy to inspect the fundus [34,35].

Genomic DNA was extracted using AutoPure LS® DNA Extractor and PUREGENE™ reagents (Gentra Systems Inc., Minneapolis, MN) from blood or saliva samples. DNA samples were also collected from 1,142 unrelated ethnically matched Caucasian healthy control participants.

### Gene screening and sequence analysis of collagen type II alpha 1 and versican genes

Primers for PCR and sequencing were designed to cover coding and untranslated gene regions, including intron–exon boundaries, using the ExonPrimer and Primer3 programs (Helmholtz Zentrum, Munich, Germany). Primers were selected to produce amplified product sizes not to exceed 900 bp for optimal sequence output and reading. Large exons or untranslated gene regions were covered with overlapping amplicons, with a minimal 50 bp of overlapped sequence. All 54 *COL2A1* exons and 15 *VCAN* exons were examined. Appendix 1 displays the optimized primer sequences used for *VCAN* and *COL2A1* screening.

Genomic DNA of two affected individuals (II:2 and II:3; Figure 1) of the study family was initially screened for sequence variations in the *COL2A1* and *VCAN* genes. The DNA of the remaining family members was subsequently screened to determine and confirm sequence variants segregation.

**Figure 1:**
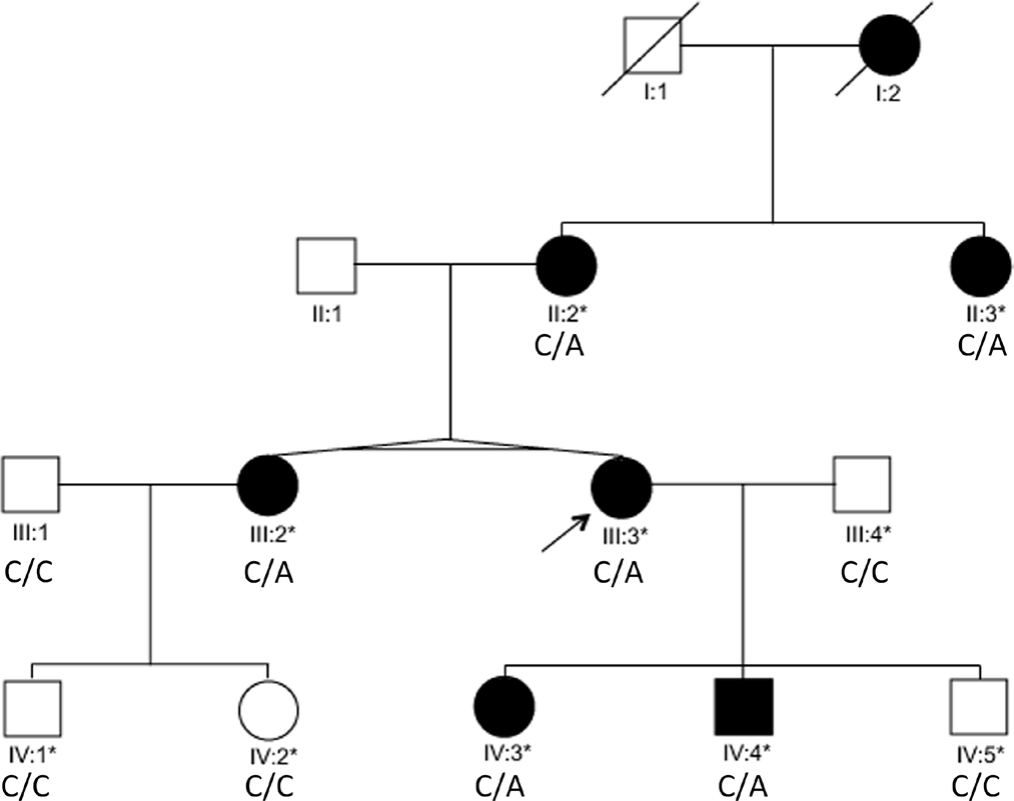
Study family pedigree. The family consisted of 14 individuals in three generations with six affected and four unaffected participants. Solid symbols indicate affected individuals. Asterisks indicate participating individuals for whom DNA was available for genomic analysis.

PCR was conducted using an Eppendorf Mastercycler Pro S® with a standard touchdown PCR protocol. PCR amplicons were visualized with 2% agarose gel electrophoresis. BigDye™ Terminator 3.1 was used to perform sequencing reactions, and ABI3730XL robotics was used to process the DNA fragments (Applied Biosystems Inc. [ABI], Foster City, CA). The Sequencher® 5.0 Software (Gene Codes, Ann Arbor, MI) was used to analyze the base pair calls. Sequences of affected and unaffected individuals were aligned to a known reference genomic sequence (UCSC Genome Browser) and compared for sequence variation. Sorting Intolerant From Tolerant (SIFT) [36] and Polymorphism Phenotyping (PolyPhen2) [37] software tools were used to predict mutational consequence of all *COL2A1* and *VCAN* variations segregating with disease.

### Genotyping

Applied Biosystems (ABI) TaqMan® SNP Genotyping assays were designed and employed to measure the allelic frequencies in 1,142 ethnically matched control DNA samples. Variants of interest were screened with PCR assay technology using TaqMan probes according to the manufacturer’s protocol (Applied Biosystems). Alleles were detected and allelic discrimination were analyzed with ABI Prism® 7900HT Sequence Detection System and ABI Sequence Detection Systems 2.4 software, respectively (Applied Biosystems). For quality control, positive and negative controls were run in the same experiment.

### Complementary deoxyribonucleic acid tissue expression

We investigated the expression of the *COL2A1* messenger ribonucleic acid (mRNA) construct(s) in fetal ocular tissues to verify the presence of type IIA and/or type IIB isoforms. Fetal ocular tissue panels not affected with disease were established internally by acquisition of whole eye globes from Advanced Bioscience Resources (Alameda, CA). Twenty-four-week fetal eyes were obtained and preserved in RNAlater® Foster City, CA within minutes of abortion and shipped overnight on ice. Whole globes were dissected the same day as they arrived, and specific ocular tissues were isolated by snap-freezing the samples and storing at −80 °C until RNA extraction. RNA was extracted from each tissue sample independently using the Ambion Foster City, CA mirVana Total RNA Extraction Kit per the protocol. The tissue samples were homogenized in Ambion’s lysis buffer using an Omni Bead Ruptor 24 Homogenizer per protocol. Reverse transcription reactions were performed with Invitrogen’s SuperScript™ III First-Strand Synthesis kit to obtain cDNA (Life Technologies, Grand Island, NY).

In-house fetal eye cDNA was amplified using primers that spanned multiple exons, not to exceed 600 bp (Appendix 1). PCRs were run using a standard protocol. Visualization of the PCR products was done on a 2% agarose gel through electrophoresis at 120 V for 50 min. Products with exon 2 were expected to amplify at 510 bp, whereas those that did not contain the exon 2 were expected to produce a product of 303 bp. Band extraction, purification, and sequencing of the bands were conducted to verify the amplicon.

## Results

### Clinical features

A three-generation family with affected member variable ocular-only diagnosis of either Stickler or Wagner syndrome was ascertained. The disease appeared to be transmitted in an AD inheritance pattern and showed variable expressivity with 100% penetrance (Figure 1). Six affected and four unaffected individuals participated in the study. Clinical data were obtained where available.

The proband, patient III:3, was a 40-year-old woman with moderate myopia and history of retinal detachment in the left eye (OS) at the age of 18 years. Periodically, she underwent prophylactic peripheral laser photocoagulation treatment bilaterally. She had cataract extraction surgery of both eyes at the age of 37 (right eye, OD) and 40 (OS). Fundus examination showed bilateral vitreous syneresis, with a vitreous membrane in the left eye, and bilateral radial extensive lattice degeneration. She had no history of systemic manifestations.

Patient III:2 was a 40-year-old woman and the monozygotic twin sister of III:3. Patient III:2 presented with moderate myopia and underwent retinal surgeries at the age of 18 (OD) and 33 (OS) for retinal detachment (OD) and epiretinal membrane and large retinal tears (OS). Subsequently, she was treated with extensive peripheral prophylactic photocoagulation. She underwent cataract extractions bilaterally at the age of 37 (OS) and 38 (OD). Fundus examination revealed bilateral radial lattice degeneration and bilateral vitreous syneresis. She had no history of systemic manifestations.

Patient II:2 was the 65-year-old mother of the twins. She had a history of bilateral congenital cataract extractions. Her fundus examination demonstrated bilateral vitreous syneresis with vitreous membranes. Patient II:3 presented with a cleft palate at an early age. Patients IV:3 and IV:4 presented with a history of retinal detachments but no systemic manifestations. We were not able to collect clinical data from the deceased individuals I:1 and I:2, although historical accounts from II:1 revealed her mother (I:2) had a history of retinal problems.

### Molecular genetic analysis

Initial genomic DNA sequencing of two affected individuals (II:2 and II:3) was conducted in the *VCAN* and *COL2A1* genes. For *VCAN*, no sequence variants segregated with the disease status were identified compared to the control DNA and to published reference sequences.

For the *COL2A1* gene, we identified 18 single nucleotide variations: one nonsense variant, one known missense variant, two known coding-synonymous variants, and 14 intronic single nucleotide polymorphisms (Appendix 1).

**Figure 2:**
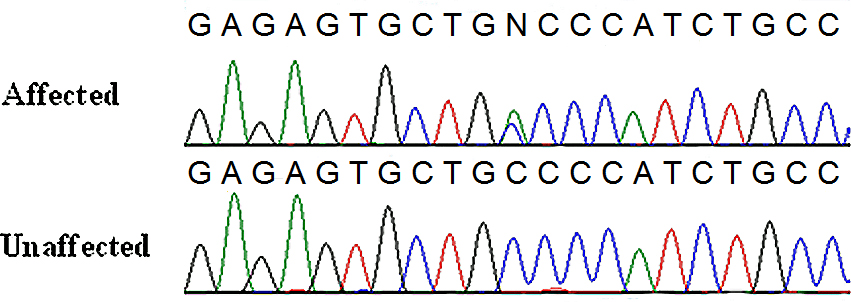
Sequence chromatogram of the Cys86X mutation in *COL2A1* exon 2. The sequence chromatogram of *COL2A1* exon 2 encompassing codon 86 demonstrates the c.258C>A mutation (GenBank NM_001844.4, +1 in cDNA numbering corresponding to the A of the methionine translation initiation codon) converting a cysteine codon to a stop codon in two affected individuals while the mutation is not present in two unaffected individuals. DNA analysis of all other affected family members cosegregated with this mutation while the mutation was not present in all unaffected family individuals.

The nonsense mutation was a C to A change (Figure 2) in exon 2 (c.258C>A; NM_001844.4), converting codon TGC for cysteine at position 86 to codon TGA for a premature stop codon (Cys86Stop; NP_001835.3). Subsequent sequencing of the remaining family members showed mutation cosegregation with the disease phenotype (Figure 2).

Genotyping was performed for the c.258C>A mutation in the *COL2A1* gene for an additional 1,142 unrelated ethnically matched controls (2,284 chromosomes). The mutation was not present in the control samples.

### Tissue expression

**Figure 3:**
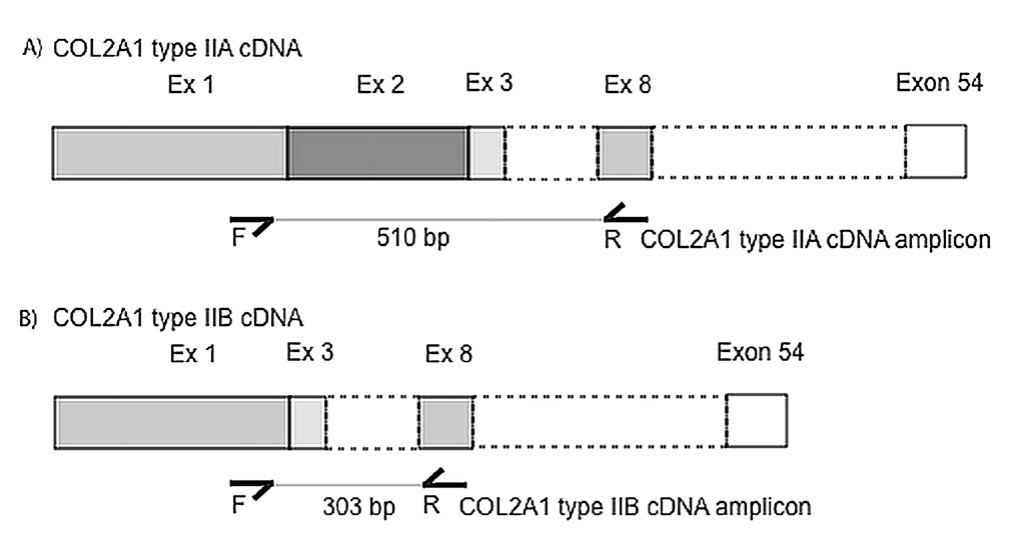
*COL2A1* cDNA structure and *COL2A1* cDNA primer design. Exon 2 undergoes tissue-dependent alternative splicing. The *COL2A1* type IIA isoform (A), expressed in the eye vitreous and in embryonic chondroprogenitor cells, includes exon 2 (Ex 2) whereas this exon is spliced in the *COL2A1* type IIB isoform (B), which is expressed by adult differentiated chondrocytes. Primers were designed to amplify both cDNA isoforms: The *COL2A1* cDNA primers span 303 bp when amplifying *COL2A1* type IIB cDNA (excluding exon 2), and 510 bp when amplified *COL2A1* type IIA cDNA (including exon 2). Ex 1, Ex 2, Ex 3, and Ex 8 depict exons 1, 2, 3, and 8.

We examined *COL2A1* expression across normal fetal eye tissues to verify the presence or absence of type IIA and/or type IIB isoforms (Figure 3). Both *COL2A1* mRNA isoforms (type IIA and type IIB) were expressed in the fetal retina/retinal pigment epithelium and choroid (Appendix 1). The *COL2A1* mRNA type IIB isoform (excluding exon 2) was expressed in the sclera, optic nerve, and cornea. Gel extraction and Sanger sequencing of the specific product bands confirmed our findings of the isoforms except for the optic nerve, where the IIA isoform was not sequenced.

## Discussion

We report a nonsense mutation in a large Caucasian family consisting of six affected individuals variably diagnosed with Stickler and Wagner syndromes. A base pair change at c.258C>A leading to a premature stop codon in exon 2 of *COL2A1* was cosegregated with the disease status. Sequencing of ocular tissues confirmed the presence or absence of exon 2, demonstrating that isoforms may be ocular tissue specific. The mutation was not present in more than 2,000 chromosomes, validating the rarity of this mutation and confirming Stickler syndrome has a predominant ocular-only phenotype.

Two striking features of Stickler syndrome are, as in our reported family, the high penetrance and variable expressivity. In the literature, the same mutation as in our family (c.258C>A; NM_001844.4) demonstrated high penetrance [3,19,20]. In families harboring alternative *COL2A1* exon 2 mutations, the ocular manifestation penetrance was also high—from 90% [20] to 100% [24,38]. The variable expressivity, even within the same family [39], contrasts with the high disease penetrance: In most reported *COL2A1* exon 2 mutations, ocular features were variable as either myopia, retinal detachment [22], or retinal degeneration [23] could be absent in affected patients. Furthermore, two common ocular features in *COL2A1* exon 2 mutations are vitreous degeneration and radial perivascular retinal degeneration [3,19,20]. Systemic manifestations were rarely associated with *COL2A1* exon 2 mutations, as manifestations were present in few cases [3,19,20,22]. In our family, only one affected individual presented with cleft palate.

Underlying causes of variable expressivity in ocular-only STL1 are still undetermined. However, in recent years the phenotypic variability of exon 2 mutations has been hypothesized to be due to degradation of mRNA by nonsense-mediated decay (NMD) or synthesis of alternatively spliced protein [21].

NMD is a regulation pathway involving the targeted degradation of mRNA that contains a premature stop codon. In this way, NMD may play a role in phenotype variability by minimizing the potential damage caused by premature termination codons [40,41]. In achondrogenesis and hypochondrogenesis caused by *COL2A1* mutations, a relationship has been proved between the severity of the phenotype and the amount of type II collagen within the cartilage extracellular matrix [42]. This implies that not only qualitative but also quantitative factors likely modulate phenotypes linked to the *COL2A1* gene [43], as in haploinsufficiency due to NMD.

Haploinsufficiency due to NMD was reported by Kaarniranta et al. [44], who found that heterozygous inactivation of *COL2A1* gene in the murine model led to structural defects and alterations that resulted from haploinsufficiency in ocular tissues containing type II collagen. These alterations included vitreous changes similar to those seen in patients with Stickler syndrome, which included reduced immunostaining of type II collagen in the vitreous and retina, in addition to reduced density of vitreous filaments in *COL2A1*+/− mutant mice [45]. Furthermore, in *COL2A1* exon 2 mutant mice with mutant allele encoding *COL2A1* mRNA without exon 2 [46], IIA+/− mutant embryos demonstrated, at an early stage, craniofacial abnormalities of truncated frontonasal structures and hypoplasia of the midface tissues. These malformations were more frequent in IIA−/− mutants. These findings are consistent with *COL2A1* type IIA mRNA expression in regions of active recruitment of cells for chondrogenesis and in areas of skeletal growth [41].

Alternatively, a second hypothesis is that nonsense-mediated altered splicing can be caused by disruption within the splicing *cis* element [21]. Minigene constructs created by McAlinden et al. demonstrated that disruptions in the enhancer sites in *COL2A1* exon 2 favor the production of the procollagen type IIB isoform. The decrease in the ratio of type IIA compared to type IIB leads to variance in expression levels, perhaps one isoform predominating over another, but the less expressed is not completely absent. These studies highlight the alternative imbalance between the two isoforms, which may have adverse effects during ocular embryogenesis [21].

Systemic manifestations associated with STL1, particularly facial development abnormalities and midline clefting as reported here (individual II:3; Figure 1), have been observed in some cases of *COL2A1* exon 2 mutations, with a frequency depending on the series, 1%, 4%, and 43% in the Donoso [3], Parma [19], and Richards [22] series, respectively. These findings are not inconsistent with exclusive expression of the longer type IIA isoform in the adult vitreous [47], as embryonic expression of this isoform has been demonstrated in chondroprogenitor tissues [48].

The Cys86Stop mutation has previously been reported in four families with Stickler syndrome whose genealogy was traced to the 16^th^ century. Subsequently, Donoso et al. identified a member of the branch that migrated to the southeastern and mid-southern United States during the 19^th^ century [3,19,20]. Some members of these families included direct descendants from the passengers of the 1620 *Mayflower* voyage [20] who arrived on the northeastern coast and then migrated to the south. Interestingly, an affected individual (III:2) reviewed her genealogy and traced her ancestry to the state of Georgia. If descendants from Donoso’s family indeed migrated south due to the Cherokee Land Grant of 1813, the similar geographical region of their cohort and ours would not exclude the possibility that the families could be related [20]. The apparent founder effect coupled with literature estimations of 50,000 to 100,000 descendants that could be related to the original family with this reported mutation demonstrates the importance of the genotype-phenotype relationship in patients with Stickler syndrome with this particular mutation [3].

The overarching similarities in phenotypes among vitreoretinal diseases make accurate diagnosis difficult clinically. Clinicians must understand and be updated on all allied conditions associated with Stickler syndrome to properly diagnose patients [47]. The characteristics of vitreous and retinal degeneration may guide molecular testing, but in case of doubt, a retinal specialist should be referred [47]. Although concentrations of exon 2 mutations are for predominantly ocular-only phenotypes, family members with mutations can still have systemic manifestations, seen in individual II:3, who presented with a cleft palate at a young age. The broad phenotypic variation seen in families with Stickler syndrome underscores the importance of using clinical and genetic testing to properly diagnose and treat patients with Stickler and Wagner syndromes.

## Appendix 1. Supplemental section contains 4 tables and 1 figure.

Supplemental section contains 4 tables and 1 figure. Table S1 contains all variants identified in COL2A1. Table S2 and S3 contains primers used for COL2A1 and VCAN gene screening. Table S4 contains primers used for cDNA amplification. Figure S1 contains PCR products of COL2A1 cDNA tissue expression. To access the data, click or select the words “Appendix 1.”
